# Liability of sepsis is hardly determined by the *COXI* variant m.6459T>C

**DOI:** 10.1111/jcmm.13957

**Published:** 2018-10-18

**Authors:** Josef Finsterer

**Affiliations:** ^1^ Krankenanstalt Rudolfstiftung Messerli Institute Veterinary University of Vienna Vienna Austria

With interest we read the article by Shen et al about seven members of a three‐generation Han Chinese family in which four members carried the mtDNA variant m.6459T>C in the *COXI* gene.^1^ The other three members of the family did not carry the mutation.^1^ Interestingly, all four mutation carriers had a history of sepsis,^1^ which was fatal for three of the mutation carriers.^1^ Mononuclear cells (B‐lymphocytes, T‐lymphocytes, monocytes) were infected with EBV. Afterwards, T‐lymphocytes and monocytes were inhibited and B‐lymphocytes stimulated to proliferate.^1^ Measurement of reactive oxygen species (ROS), the mitochondrial membrane potential, the apoptosis status, and the mitochondrial ATP level revealed that mutation carriers had increased ROS and apoptosis and decreased mitochondrial membrane potential and ATP‐levels compared to non‐mutation carriers.^1^ The authors concluded that m.6459T>C mutation carriers are prone to experience sepsis. We have the following comments and concerns.

We should be informed at which time point of the history blood was taken from the seven probands. Since three of the mutation carriers had died already, blood from these three subjects must have been drawn for the investigation prior to death. Was blood drawn during the infection in all four patients experiencing sepsis or was it taken during a period of healthiness? The time point at which blood was drawn is crucial as lymphocytes may have different potentials to react during infection and during health.^2^

As the pedigree are small and the three affected males (II‐1, II‐2. II‐4) did not have children the occurrence of sepsis in the mutation group could have followed also an autosomal dominant trait of inheritance. Thus, also other hereditary immunosufficiency syndromes need to be excluded, such as immunodeficiency due to *TWEAK* mutations^3^ or hyperimmunoglobulin E syndrome, Were the levels of immunoglobulins normal prior to sepsis in the four mutation carriers? Was the status of the cellular immune system normal prior to the infection?

A further shortcoming of the study is the low number of subjects in the mutation and non‐mutation group.^1^ Only four patients constituted the mutation group and only three patients the non‐mutation group.^1^ The low number of patients represents a strong limitation for statistical comparisons, as both groups are heavily underpowered.

Though we agree that immune cells may be affected in patients with a mitochondrial disorder (MID),^4^, ^5^ MIDs usually manifest as a multiorgan syndrome, affecting the brain, muscles, peripheral nerves, eyes, ears, endocrine organs, the myocardium, the gastrointestinal tract, the lungs, the kidneys, the blood cells, the cartilage, and the skin in addition to the immune cells.^6^ Thus, it is important to prospectively investigate carriers of mtDNA mutations for clinical or subclinical involvement of the other organs. We also should be informed if biochemical investigations of the muscle or of skin fibroblasts or immunohistochemistry revealed COX‐deficiency, respectively, reduced activity of the complex four of the respiratory chain.

Since phenotypic manifestations of mtDNA variants strongly depend on the mutation load of the variant (heteroplasmy rate), we should be informed about differences of the mutation loads between the four mutation carriers. Heteroplasmy rates not only in lymphocytes but also in hair follicles, buccal mucosa cells, skin fibroblasts, muscle, and urinary epithelial cells should be compared. Differences of ROS, apoptosis frequency, membrane potential, and ATP levels between nutation carriers and controls can be reliably assessed only if heteroplasmy rates are determined and compared between the included subjects.

As MIDs frequently manifest with lactic acidosis, it would be interesting to know if there were any differences between the mutation and non‐mutation group with regard to lactate concentrations in the serum and brain.

Overall, we doubt that the m.6459T>C variant was responsible for sepsis in the four mutation carriers described. Arguments against the speculation that the variant is responsible for the liability to infections are that the pathogenicity of the variant was not confirmed, that heteroplasmy rates were not considered, and that manifestations in organs other than immune cells were not described.

## References

[1] Shen X , Han G , Li S , et al. Association between the T6459C point mutation of the mitochondrial MT‐CO1 gene and susceptibility to sepsis among Chinese Han people. J Cell Mol Med 2018 Sep 11. 10.1111/jcmm.13746. PMC6201344 30207067

[2] Ourives SS , Borges QI , Dos Santos DSA , Melo ECM , de Souza RM , Damazo AS . Analysis of the lymphocyte cell population during malaria caused by Plasmodium vivax and its correlation with parasitaemia and thrombocytopaenia. Malar J. 2018 Aug 20;17(1):689 10.1186/s12936-018-2443-x. PMC6102853 30126413

[3] Wang HY , Ma CA , Zhao Y , et al. Antibody deficiency associated with an inherited autosomal dominant mutation in TWEAK. Proc Natl Acad Sci USA. 2013;110:5127‐690. 23493554 10.1073/pnas.1221211110 PMC3612633

[4] Finsterer J , Zarrouk‐Mahjoub S . Affection of immune cells by a C10orf2 mutation manifesting as mitochondrial myopathy and transient sensory transverse syndrome. Acta Neurol Belg. 2017;117:969‐970. 28735506 10.1007/s13760-017-0821-8

[5] Galassi G , Maggi L , Lamantea E , Ariatti A , Malagoli M . C10ORF2 mutation associated with progressive external ophthalmoplegia and clinically isolated syndrome. Acta Neurol Belg. 2017;117:947‐949. 28528470 10.1007/s13760-017-0793-8

[6] Finsterer J , Zarrouk‐Mahjoub S . Mitochondrial multiorgan disorder syndrome score generated from definite mitochondrial disorders. Neuropsychiatr Dis Treat. 2017;13:2569‐2579. 29062232 10.2147/NDT.S149067 PMC5638572

